# Posteclampsia Sudden Cardiac Arrest (SCA): A Rare Etiology

**DOI:** 10.1155/2020/8862839

**Published:** 2020-09-08

**Authors:** Nissar Shaikh, Shoaib Nawaz, Arshad Chanda, Seema Nahid, Muhmmad Zubair, Firdous Ummunnisa

**Affiliations:** ^1^Department of Anesthesia/SICU and Perioperative Medicine, Hamad Medical Corporation, Doha, Qatar; ^2^Department of OBGY, Hamad Medical Corporation, Doha, Qatar

## Abstract

Eclampsia is associated with high maternal and fetal morbidity and mortality. The mortality in eclampsia is reported to be secondary to cerebrovascular accidents, neurogenic pulmonary edema, or acute kidney injury leading to cardiac arrest. A rarely reported etiology is sudden cardiac arrest (SCA) immediately after the seizure activity. We report a case of morbidly obese multigravida, complicated into postnatal eclampsia developing postseizure SCA due to apnea. *Case*. A 35-year-old woman in 38 weeks of gestation presented to the women's hospital emergency with hypertension and proteinuria and had lower section caesarean section under epidural anesthesia and required labetalol infusion. She developed convulsions in the 1st postoperative day, and she was started on magnesium sulphate therapy. After a few minutes, the patient had a 2nd episode of convulsions, apnea, cyanosis, and cardiac asystole requiring cardiopulmonary resuscitation and spontaneous circulation returned in 3 minutes. Her endotracheal intubation was difficult, but we succeeded in the 2nd attempt. She was sedated, ventilated, and required noradrenaline to maintain hemodynamics. Her ECG, echocardiogram, cardiac biomarkers, CT chest/brain, and serum magnesium levels were within normal range. The patient was weaned from vasopressor and ventilator by day 2 and extubated. She became awake; labetalol and magnesium sulphate infusions were stopped by day 3. The patient was transferred to the ward on day 5; from there she was discharged home on day 8 on oral labetalol. She was followed up in an outpatient clinic after 4 weeks and remained comfortable, and blood pressure was controlled with tablet labetalol and repeat echocardiogram was normal. *Conclusion*. Eclampsia patients can have apnea after seizures, progressing to SCA.

## 1. Introduction

Eclampsia is the occurrence of seizures after 20 weeks of pregnancy up to the 10^th^ postpartum day commonly associated with preeclampsia, although 40% of eclampsia patients will not show any premonitory symptoms and signs [1, 2]. The overall incidence of eclampsia is showing increasing trends with incidence from the world and Middle East region reported to be 3.2/10000 deliveries [2]. Eclampsia had remained a significant cause for maternal and fetal mortality over decades [1, 2].

Eclampsia mortality and cardiac arrest are reported to be secondary to cerebrovascular accidents, posterior reversible encephalopathy syndrome, and neurogenic pulmonary edema [1–3]. Sudden cardiac arrest (SCA) in eclampsia is rarely reported [4]. We report a case of SCA in a morbidly obese eclampsia patient due to respiratory apnea.

## 2. Case

**Figure 1:**
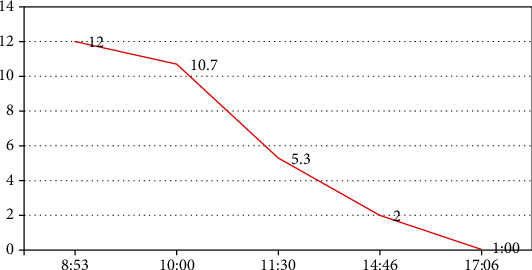
Serum lactate levels (mmol/L).

A 35-year-old morbidly obese (BMI 61) patient (there was no history of apnea attack in her usual days), gravida 6 para 4 with 4 previous lower section caesarean sections (LSCS), in 38 weeks of gestation, presented to the women's hospital emergency with hypertension (blood pressure (BP) 160/96 mmHg) and proteinuria (2+). Labetalol infusion was started to control the hypertension. Enoxaparin was started for DVT (deep venous thrombosis) prophylaxis. She had LSCS and tubal ligation under epidural anesthesia. The perioperative period was uneventful, and she was continued on labetalol infusion and epidural analgesia in the HDU (high-dependency unit). Her complete blood count, haematocrit, and serum electrolytes were within normal range. On postoperative day 1, the patient was fully awake and stable. Labetalol infusion was stopped and shifted to oral labetalol, and epidural catheter was removed. Suddenly, she had tonic-clonic seizures lasting for 1 minute with hypertension (BP = 200/110). She was diagnosed to have postpartum eclampsia and was given a loading dose of magnesium sulphate (2 g) (MgSO_4_), and infusion of MgSO_4_ and labetalol started. The patient was arousable, and BP decreased to around 140/80 mmHg. After 30 minutes, she had a 2^nd^ episode of seizures, she was already on oxygen supplementation, and oral airway was inserted and tilted to one side. The seizure activity subsided in one minute. We noticed that she was apnoeic, cyanosed, and went into cardiac asystole. Immediate CPR (cardiopulmonary resuscitation) started with bag-mask ventilation. Code blue team was activated. She was difficult to intubate due to thick, short neck and poor oral hygiene with loose teeth. ROSC (return of spontaneous circulation) was achieved in 3 minutes of CPR, but the patient was not awake; it was decided to intubate her; the laryngeal mask was initially inserted, and under the effect of propofol, fentanyl, and rocuronium, intubation with video laryngoscopy was tried but failed. Subsequently, in the 2^nd^ attempt with direct laryngoscopy, we were able to intubate her trachea successfully. There was no edema of the vocal cords or surrounding structures. On chest auscultation, there were no added (rhonchi or crept) sounds and equal air entry. The patient was shifted to an intensive care unit (ICU) bed and connected to a ventilator with sedation and analgesia, central venous catheter and arterial lines were inserted, her pupils were equal and constricted, oxygen saturation was 96 to 100%, blood pressure was lower (88/52 mmHg), labetalol infusion was stopped, and she was started on noradrenaline and continued on MgSO_4_ infusion after the 2^nd^ bolus of 2 grams. Her laboratory workup showed normal complete blood count; coagulation profiles included normal antithrombin III levels, serum electrolytes, and magnesium level which was 1.37 mmol/L. Her serum lactate was elevated (12.0 mmol/L), and urine output was around 50 mL/h and it was concentrated requiring fluid boluses apart from baseline infusion. Her chest X-ray showed that there was no abnormality; computerized tomography (CT) brain did not show any pathological findings, and CT angiogram ruled out pulmonary embolism. The patient started to localize pain after 16 hours on ventilator; serum lactate became normal (Figure 1), and urine output improved. We could wean off noradrenaline in the next few hours. By postoperative day 2, the patient was fully awake and hemodynamically stable; spontaneous awakening (SAT) and breathing (SBT) trials were successful; she was extubated and started on supplemented oxygen through nasal cannula and injected with fentanyl intravenously for analgesia. The patient was awake, and she was maintaining oxygen saturation. After 4 hours of extubation despite pain control, she was hypertensive and was restarted on labetalol infusion and continued with MgSO_4_ infusion. The patient remained stable and awake. On day 3, invasive lines were removed, labetalol and MgSO_4_ infusions were stopped, and oral labetalol 200 mg three times per day was started. The patient was transferred to the ward on day 4; from there she was discharged home to be followed in an outpatient clinic. She was comfortable during the follow-up after 4 weeks, she remained on oral labetalol, and her repeat echocardiogram was normal.

## 3. Discussion

Sudden cardiac arrest (SCA) after seizure activity is defined as the absence of pulse or cardiac arrest within one hour of the event [5]. SCA following seizures is not reported in the literature. The frequent morbidities in eclampsia patients are abruptio placenta, pneumonia, HELLP syndrome, acute kidney injury, pulmonary edema, cerebrovascular accidents, or PRES (posterior reversible encephalopathy syndrome), and most of the mortality and secondary cardiac arrest are reported secondary to PRES, cerebrovascular accidents, pulmonary edema, or acute kidney injury [1–3, 6]. We can find an older case report of SCA in an eclampsia patient due to magnesium sulphate (MgSO_4_) overdose; this report was from the initial days of using MgSO_4_ in eclampsia patients. Presently, MgSO_4_ is commonly used as an anticonvulsant in eclampsia patients due to efficacy, safety, and cost-effectiveness [2]. In our patient, serum MgSO_4_ levels were not high and they were slightly lower than the protocol range (2-3 mmol/L).

The epilepsy literature describes well the occurrence of SCA after tonic-clonic convulsions, commonly due to the various cardiac arrhythmias ranging from bradycardia, asystole, and atrial fibrillation to ventricular fibrillations in the postictal period [7]. Our patient was on continuous ECG monitoring, and there were no cardiac arrhythmias prior to asystole.

Central apnea syndrome is characterised by cessation of spontaneous breathing drive in the postictal states [7, 8]. Seizure activity is known to cause central apnea by direct flow of electric discharge to the respiratory centres [7, 8]. van der Lende et al. found only 8 cases of central apnea syndrome following the seizure activity before proceeding into asystole and SCA [7]. Recently, Seo and Sung reported a case of near-miss unexpected death in an epileptic patient due to postconvulsion central apnea [8]. We witnessed in our patient apnea and cyanosis prior to the cardiac arrest; hence, it was postseizure cessation of breathing leading to SCA. Our patient was obese with high body mass index, prone to have obstructive sleep apnea, which may be an added risk along with the central cause for cessation of spontaneous breathing activity.

The concluding lines from our case report are that eclampsia patients can have apnea after tonic-clonic convulsions, which can complicate into SCA, and the airway can be difficult to secure. A multidisciplinary team approach is essential for the better outcome in these situations.

## Data Availability

The data used to support the findings of this study are available upon request.
